# In the June 2013 issue

**DOI:** 10.1590/S1980-57642013DN70200001

**Published:** 2013

**Authors:** Ricardo Nitrini

**Affiliations:** Editor-in-Chief

In this issue we are publishing two reviews and ten original papers, one of which is a systematic review of the literature, and the abstracts of the World Congress on Brain, Behavior and Emotions.

**O'Connor et al.** reviewed the literature on non-pharmacological interventions to manage symptoms of frontotemporal dementia (FTD). A few studies have been published but more rigorous approaches are needed. A promising intervention, the Tailored Activities Program, has yet to be trialled in FTD. This approach incorporates the concepts of using individualised activities with patients in conjunction with carer education.

**Oliveira and Oliveira** performed a literature review highlighting main neuropsychological findings of basal ganglia calcification. Disturbances of selective attention, verbal perseveration and declarative memory have been described in case reports. Basal ganglia calcification (BGC) in its idiopathic or secondary form has been classically labeled as a typical type of subcortical cause of dementia with cognitive and behavioral symptoms including mood disorders and psychosis. Further studies are necessary to investigate this group of diseases presenting with basal ganglia calcification.

**Müller and de Salles** carried out a systematic review on the role of the right cerebral hemisphere in semantic priming effects (SPEs) through the investigation of studies of patients affected by right hemispheric strokes. Difficulties in SPEs were shown in almost half of the included studies, showing that this ability should be evaluated in individuals suffering a stroke in the right hemisphere to provide appropriate treatments.

**Wajman and Bertolucci** performed the translation and back-translation of the short version of the severe impairment battery (SIB-8) and applied it to 95 patients with moderate to severe dementia due to Alzheimer's disease (AD). Their findings indicated that the SIB-8 retained its original characteristics in the Brazilian population and is a useful tool for the evaluation and prospective comparison of AD patients in advanced stages.

**Caldas et al.** evaluated the prevalence of neuropsychiatric symptoms (NPS) in the different stages of dementia severity to verify whether there is a trend for increased prevalence of some NPS from the mild to advanced stages of dementia. In a cross-sectional study of 124 patients attending an outpatient dementia clinic, the authors found apathy (75%) and irritability (66.9%) as the most frequent NPS when using the Neuropsychiatric Inventory. Only apathy and aberrant motor behavior showed increased prevalence in more advanced stages of dementia severity.

**Gorzoni et al.** investigated the pharmacologic drug consumption by 29 demented and 21 non-demented elderly patients attending an outpatient geriatric clinic. There was no difference between demented and non-demented patients regarding the pattern of drug consumption although the mean number of simultaneously used drugs was high in both groups.

**Sobral and Paúl** investigated the association of education and particularly, of participation in leisure activities, with the cognitive and functional ability of AD patients and with the course of the disease. Functional and neuropsychological abilities of 120 outpatients with probable AD were evaluated at baseline, 36 and 54 months. The participants with higher participation in leisure activities exhibited better results on cognitive and functional tests than those with lower participation. Their results also suggest a slower rate of disease progression in patients with a higher level of participation in leisure activities throughout their lives.

**Miranda et al.** evaluated the rate of response to cholinesterase inhibitors (ChEI) in 71 patients with AD who were assessed before starting treatment by a comprehensive neuropsychological battery. Good response was defined by a gain of ≥2 points on the MMSE after three months of treatment in relation to baseline. The good response rate at three months was 31.0% overall, being 37.2% and 21.4% in mild and moderate dementia, respectively. The rate of good clinical response to ChEI was higher than previously-reported levels. Moderate dementia patients also had a significant reduction in hallucination, agitation and dysphoria scores on the NPI.

**Satler and Tomaz** evaluated the relationship between the presence of anosognosia symptoms and cognitive domains, functional abilities, and neuropsychiatric symptoms in Alzheimer's disease. Twenty-one AD patients were compared to 22 elderly controls using a comprehensive neuropsychological battery as well as the Neuropsychiatric Inventory and Functional Activities Questionnaire. The best predictors for self-awareness were executive functions, particularly for behavioral changes.

**Shigaeff et al.** compared the cognitive status of 25 elderly patients with metabolic syndrome (MetS) to 24 elderly controls. All subjects underwent a global geriatric and neuropsychological assessment. There was no statistically difference between the groups. The results obtained in the study showed that MetS was not associated with cognitive impairment in this population, but further prospective studies are necessary to investigate the influence of MetS on cognitive performance among elders.

**Oliveira et al.** evaluated the prevalence of brain calcifications in 1,898 consecutive patients who had undergone a cranial computed tomography scan. All scans were reviewed by the same radiologist. Brain calcifications were described in 333 subjects (17.54%), and among parenchymal calcifications, basal ganglia was the main site with a prevalence of 2.42%, followed by parietal (1.36%), occipital (0.52%), frontal (0.42%), cerebellar (0.36%) and temporal (0.26%) lobes.

**Paiva et al.** investigated the advantages of using a magnetic field of 3.0T in the measurement of brain metabolites in a typical clinical routine setting. Single voxel spectra were acquired from the posterior cingulate cortex in 26 healthy subjects. Each subject was scanned consecutively at 1.5T and 3.0T in a randomly distributed order. Even though the theoretically predicted 100% improvement in SNR and spectral resolution cannot be achieved in practice, the benefits of higher field strengths for MRS are clear. Future comparative studies will be necessary to refine the metabolite thresholds for early detection and quantification of distinct neurological and psychiatric disorders using 3.0T MRS.

Ricardo Nitrini Editor-in-Chief

